# The Concentration of 8-Hydroxy-2′-Deoxyguanosine in Plasma During the Menstrual Cycle in Young Japanese Women

**DOI:** 10.1089/whr.2021.0067

**Published:** 2022-02-22

**Authors:** Kaori Yama, Honoka Shinbo, Yuka Fujikane, Chiaki Mikami, Maiko Machida, Jun Miura

**Affiliations:** Department of Pharmacy, Hokkaido University of Science, Sapporo, Japan.

**Keywords:** premenstrual syndrome, 8-hydroxy-2′-deoxyguanosine, oxidative stress, menstrual cycle, depression

## Abstract

***Background:*** The exact pathophysiology of premenstrual syndrome (PMS) and premenstrual dysphoric disorder (PMDD) is still unknown. This study aimed to investigate the concentration of 8-hydroxy-2′-deoxyguanosine (8-OHdG) in plasma in relation to the menstrual cycle and the severity of premenstrual symptoms in young Japanese women.

***Methods:*** The study included 21 healthy Japanese women 19–24 years of age. Fourteen women had no or mild PMS [PMS (−)], while five women had moderate to severe PMS and two women exhibited PMDD [PMS (+)]. The concentration of 8-OHdG in plasma was measured by means of high-performance liquid chromatography–electrochemical detector. The Center for Epidemiologic Studies Depression (CES-D) scale was used to evaluate the depressive tendency.

***Results:*** The concentration of 8-OHdG before menstruation was significantly higher than that after menstruation in total subjects (*p* = 0.04). In the PMS (+) group, the 8-OHdG concentration before menstruation was higher than that after menstruation (*p* = 0.02). Moreover, the PMS (+) group showed a higher 8-OHdG concentration compared with the PMS (−) group before menstruation (*p* < 0.01), as well as higher CES-D scores compared with the PMS (−) group both before and after menstruation (*p* < 0.01).

***Conclusions:*** These results suggested that the oxidation of DNA occurred before menstruation in PMS. The depression was associated with PMS symptoms both before and after menstruation in patients with PMS. Oxidation of DNA due to oxidative stress and depression in PMS patients may be involved in the pathogenesis of PMS. Clinical Trial Registration number 15-02-011.

## Introduction

According to population-based studies, 30%–40% of women in the reproductive period meet the diagnostic criteria for premenstrual syndrome (PMS).^1,2^ As defined in the tenth revision of the International Classification of Diseases (ICD-10), PMS is “characterized by a group of symptoms, such as depression, anxiety, irritability, fatigue, breast tenderness, and sleep disturbance, which start 1 to 2 weeks before menstruation and disappear after menstruation starts.”^3^ A severe subtype of PMS, where affective symptoms predominate, has been classified as premenstrual dysphoric disorder (PMDD) and categorized as a depressive disorder in the Diagnostic and Statistical Manual of Mental Disorders Fifth Edition (DSM-5).^4^

It has been shown that PMS, especially PMDD, has a negative influence on personal relationships; moreover, in PMS, work attendance and productivity, as well as health care costs and utilization, are negatively affected.^1^ Understanding the pathophysiology of PMS and PMDD is beneficial for the management of these negative aspects. However, the exact pathophysiology of PMS and PMDD is not yet known. Elucidation of the pathogenesis and, consequently, the establishment of effective treatments for PMS and PMDD are highly desired.

Several possible causes of PMS and PMDD have been suggested, including decreased progesterone level and secretion of the neurotransmitters such as serotonin or ɤ-aminobutyric acid.^5,6^ Possible risk factors associated with PMS and/or PMDD include inappropriate stress management, lack of exercise, and dietary alterations, such as alterations in vitamins B6 and E levels and insufficient levels of calcium and magnesium.^5,6^ Treatment with α-tocopherol (vitamin E), a representative antioxidant, might be effective for PMS and PMDD.^7^ We have previously reported that the biological antioxidant potential was significantly lower in the PMS group before than after menstruation, indicating that antioxidant capacity fluctuates through the menstrual cycle in the same subjects with PMS.^8^

Therefore, it can be speculated that controlling the fluctuation of antioxidant capacity could help to cope with PMS. In addition, fluctuating oxidative stress might be related to the occurrence of PMS and PMDD. To our knowledge, fluctuation in antioxidant capacity in the same subject with PMS has been investigated by two groups. Duvan et al. have reported that total antioxidant capacity in serum on day 21 was significantly lower than that on day 3, which might be in the menstruation phase.^9^ In contrast, Kalia et al. have found no difference in antioxidants between the premenstrual phase and after the menstruation.

Oxidative stress induces the oxidation of lipids, proteins, and DNA. It has been reported that the level of malondialdehyde (MDA) as a marker of lipid peroxidation did not differ significantly between PMS patients and controls.^10,11^ However, among 20 previously described DNA oxidation products, 8-hydroxy-2′-deoxyguanosine (8-OHdG) has been most often studied because of its sensitivity and mutagenic potential^12,13^; it is also considered an oxidative stress biomarker. An increase in the concentration of 8-OHdG has been observed in psychiatric diseases, including depression, bipolar disorder, and anxiety disorders.^14,15^ However, it is not clear whether oxidative stress-induced DNA oxidation is involved in the pathogenesis of PMS and PMDD. In addition, Duvan et al.^9^ and our previous study suggested that oxidative stress and antioxidant capacity may fluctuate during the menstrual cycle.

Nevertheless, 8-OHdG has not been investigated in the same subjects before and after menstruation in relation to PMS and PMDD. Therefore, in this study, we analyzed the concentration of 8-OHdG in plasma in relation to the menstrual cycle and to the severity of the premenstrual symptoms in young Japanese women.

## Materials and Methods

### Chemicals

We obtained 8-OHdG from Sigma (St. Louis, MO). In addition, we obtained *dl*-isoproterenol hydrochloride, which was used as an internal standard (IS); sodium dihydrogen phosphate dehydrate; and disodium hydrogen phosphate 12-water from Wako Pure Chemical Industries (Osaka, Japan). We purchased ethylene diamine tetra-acetic acid disodium salt (EDTA-2Na) from Dojindo Laboratories (Kumamoto, Japan). Methanol of chromatographic grade was produced by Kanto Chemical Co., Inc., (Tokyo, Japan). Ultra-pure water was produced using the Milli-Q water purification system (Millipore, Bedford, MA). Other chemicals and solvents were of high-performance liquid chromatography (HPLC) grade or reagent grade quality.

### Standard preparation

First stock standard solutions (1 mg/mL) of 8-OHdG and IS were prepared in ultra-pure water and stored at −80°C. The assay standards for each assay were prepared fresh by diluting the stock standard solutions with the mobile phase.

### Participants

We recruited healthy Japanese females with a menstrual cycle of 28 ± 5 days between February and April 2016; the subjects were students at the Hokkaido University of Science. None of the subjects was taking medications or food supplements. They were all nonsmokers and did not have any history of serious diseases. We excluded the subjects with abnormalities in hemostatic function and coagulability.

The purpose and design of this study were explained to the subjects, and they gave their written, informed consent. The study was carried out in accordance with the Declaration of Helsinki and approved by the Ethics Committee of Hokkaido Pharmaceutical University (IRB Approval No. 15-02-011).

We included 21 healthy Japanese women 19–24 years of age. Progesterone levels were measured using electrochemiluminescence immunoassay (SRL, Inc., Sapporo, Japan) to confirm the luteal phase. The reference values for progesterone in the luteal phase were >2.05 ng/mL. We excluded participants with progesterone levels below 2.05 ng/mL from the analysis.

### Plasma sampling

About 5 mL of whole blood was collected between 8:30 and 10:30 am into tubes containing 0.01 mM of EDTA-2Na, and plasma was obtained. The samples of plasma were stored at −80°C until analysis.

### Assessment of PMS

To assess psychological and physical symptoms before menstruation, we applied the PMDD scale, which is a Japanese modification^16^ of the premenstrual symptoms screening tool.^17^ The PMDD scale encompasses sections A and B. Specifically, the questions in section A focus on the symptoms, such as depressive mood, anxiety, tearfulness, anger, decreased interest, poor concentration, fatigability, overeating, sleep disturbances, and feeling out of control, as well as physical symptoms. Section B addresses the interference with activities at the workplace or school; housework; and relationships with coworkers, family, and friends. The rating of the severity of symptoms or interference was as follows: “not at all,” “mild,” “moderate,” or “severe.”

PMDD was determined on the basis of a participant having answered “severe” to at least one of the first four items in section A, “moderate” or “severe” to at least five items in total in section A, and “severe” to at least one of the items in section B. In addition to PMDD, moderate to severe PMS was defined if a participant answered “moderate” or “severe” to at least five items in section A, at least one of which had to be within the first four items, and answered “moderate” or “severe” to at least one of the items in section B. The remaining participants were regarded as having mild PMS or no PMS.

### Apparatus

The Eicom PC-300 HPLC system (Eicom, Kyoto, Japan) was used, coupled with the Eicom ECD-300 electrochemical detector (ECD) with a glass carbon cell WE-GC. The system was operated in the d.c. mode at +500 mV versus an Ag/AgCl electrode. Chromatograms were recorded and evaluated using a Power Chrom System EPC-300 (Eicom).

### Chromatographic analysis of 8-OHdG in plasma sample

The HPLC separation condition used was based on the previous work described by Yohei et al.^18^ and Koide et al.^19^ In this study, the HPLC–ECD analysis method was slightly modified. The eluate and the plasma level of 8-OHdG were measured using the HPLC–ECD. The separation of 8-OHdG was carried out on Develosil RPAQUEOUS-AR-5 Column C30 (250 × 0.6 mm I.D., 5 μm) analytical column (Nomura Chemical Co., Ltd., Aichi, Japan); the mobile phase used for isocratic elution of 8-OHdG and IS consisted of a mixture of 0.1 M sodium phosphate buffer (pH 6.0) and methanol (90:10, v/v), and 5 mg/L EDTA. The solution was vacuum filtered through a 0.22 μm cellulose acetate filter and ultrasound bath for 30 min.

The flow rate was 0.5 mL/min, and the temperature was maintained at 40°C. The optimal detection was achieved at 500 mV potential. The injection volume was 20 μL, and the retention time of the eluted 8-OHdG and IS was 12 min and 8 min, respectively. There was a high correlation between the concentration of 8-OHdG and the ratio of 8-OHdG and IS peak-area (*r*^2^ = 0.999).

8-OHdG and IS were added to the obtained plasma at a final concentration of 1 ng/mL and centrifuged (15000 rpm, 4°C, 30 min) using an Amicon Ultra-0.5 Centrifugal Filter Devices (30K) for ultrafiltration (Millipore). The correlation coefficient was 0.999. The lower limit of detection of 8-OHdG was 0.025 ng/mL. The averages of 8-OHdG and IS recoveries were 113.2% and 101.4%, respectively.

### Assessment of depression

We adopted the Center for Epidemiologic Studies Depression (CES-D) scale^20^ to evaluate the level of depressiveness in the study subjects both before menstruation and after menstruation. The CES-D scale is a four-grade (0–3) scale, including 20 questions for self-assessment of depression; we used the Japanese version of the CES-D scale.^21^ The total score on the CES-D scale is between 0 and 60. Subjects with a total score of ≥16 tend to be more depressive, especially those with higher scores.

### Statistical analyses

We recorded main demographic characteristics, including age, menstrual cycle, and days of collecting blood samples; these characteristics were compared between the subjects with PMS and those without PMS symptoms using the *t* test. The CES-D scores were expressed as median (range); since these data did not follow the normal distribution, they were analyzed by nonparametric tests. Specifically, data before and after menstruation were compared using the Wilcoxon signed-rank test. The data were compared between the subjects with PMS symptoms and those without PMS symptoms by the Mann–Whitney *U* test. The correlation of 8-OHdG and CES-D score was analyzed using a Spearman's rank correlation coefficient. All the analyses were conducted in Bell Curve Excel statistics for Windows (SSRI, Tokyo, Japan). The significance level was set at a *p*-value of 0.05 and lower.

## Results

### Background of the study subjects

Twenty-one Japanese women were offered to participate and none of them met the exclusion criteria. They were 19–24 years of age (average 22 ± 1 years). The mean duration of the menstrual cycle was 30.1 ± 3.1 days. The blood samples were collected 5.6 ± 2.9 days before the start of menstruation and 7.4 ± 1.6 days after. Five women had moderate to severe PMS, while two women had PMDD (PMS (+) group). Fourteen women had no or mild PMS [PMS (−) group]. The mean age of PMS (+) and PMS (−) groups was 21 ± 2 years and 22 ± 1 years, respectively. The mean duration of the menstrual cycle was 30.6 ± 4.4 days in the PMS (+) group and 30.0 ± 2.4 days in the PMS (−) group. There were no significant differences between the groups in age, duration of the menstrual cycle, and timing of blood sampling (Table 1).

**Table 1.** Background of Study Subjects

	Total (*n* = 21)	PMS (+) (*n* = 7)	PMS (−) (*n* = 14)
Age (years)	22 ± 1	21 ± 2	22 ± 1
Menstrual cycle (days)	30.1 ± 3.1	30.6 ± 4.4	30.0 ± 2.4
Date of collecting blood before menstruation (days)	5.6 ± 2.9	5.7 ± 2.8	5.7 ± 3.1
Date of collecting blood after menstruation (days)	7.4 ± 1.6	7.3 ± 1.1	7.5 ± 1.9

Data are presented as median (IQR).

None or mild PMS; PMS (−).

Moderate to severe PMS, and PMDD; PMS (+).

IQR, interquartile range; PMDD, premenstrual dysphoric disorder; PMS, premenstrual syndrome.

### 8-OHdG levels

The plasma 8-OHdG concentration before menstruation was significantly higher than that after menstruation in total sample (*p* = 0.04) (Table 2). The 8-OHdG concentration before menstruation was higher than that after menstruation in the PMS (+) group (*p* = 0.02), but not in the PMS (−) group (*p* = 0.69). The concentration of 8-OHdG showed higher values in the PMS (+) group than in the PMS (−) group before menstruation (*p* < 0.01), but did not differ between the groups after menstruation (*p* = 0.53).

**Table 2.** 8-OHdG Levels in the PMS (+) and PMS (−) Groups Before and After Menstruation

8-OHdG levels (ng/mL)	Before menstruation	After menstruation	*p* ^*^
PMS (+) (*n* = 7)	0.78 (0.69–4.18)	0.71 (0.07–0.76)	0.02^*^
PMS (−) (*n* = 14)	0.31 (0.00–3.99)	0.27 (0.00–1.61)	0.69
Total (*n* = 21)	0.57 (0.00–4.18)	0.43 (0.00–1.61)	0.04^*^
*p* 0.53^#^	<0.01^#^	0.53	

Data are presented as median (min–max).

^*^ *p* < 0.05, before menstruation versus after menstruation.

^#^ *p* < 0.05, PMS (+) versus PMS (−).

8-OHdG, 8-hydroxy-2′-deoxyguanosine.

### CES-D score

The CES-D scores did not change in relation to menstruation in total sample (Table 3). However, in the PMS (+) group, there was a tendency for higher CES-D scores before menstruation compared with those after menstruation (*p* = 0.06). In contrast, no change was observed in the PMS (−) group (*p* = 0.83). The PMS (+) group, both before and after menstruation, showed significantly higher CES-D scores compared with the PMS (−) group (*p* < 0.01 for both before and after menstruation). In addition, there were no significant correlations between 8-OHdG concentration and CES-D score in the total sample and PMS (+) and PMS (-) groups (data not shown).

**Table 3.** CES-D Scores in the PMS (+) and PMS (-) Groups Before and After Menstruation

CES-D score (point)	Before menstruation	After menstruation	*p*
PMS (+) (*n* = 7)	41 (16–50)	26 (9–42)	0.06
PMS (−) (*n* = 14)	11 (4–21)	12 (5–29)	0.83
Total (*n* = 21)	17 (4–50)	16 (5–42)	0.22
*p* ^#^	<0.01^#^	<0.01^#^	

Data are presented as median (min–max).

^#^ *p* < 0.05, PMS (+) versus PMS (−).

CES-D, Center for Epidemiologic Studies Depression.

## Discussion

The plasma concentration of 8-OHdG before menstruation was significantly higher than after menstruation in the total sample (Table 2) and in the PMS (+) group, but not in the PMS (−) group. Subjects with PMS exhibited higher concentrations of 8-OHdG compared with those in the PMS (−) group before menstruation. However, the two groups did not show differences in 8-OHdG concentration after menstruation. These results suggested that DNA was oxidized before menstruation in subjects with PMS. Oxidation of DNA due to oxidative stress in PMS patients may be involved in the pathogenesis of PMS, suggesting that 8-OHdG might be useful as an indicator of PMS.

Our previous study indicated a decrease in the antioxidant capacity before menstruation in subjects with PMS^8^; these results imply that the increased oxidative stress and decreased antioxidant capacity in patients with PMS produce an imbalance in oxidative status before menstruation, which may be involved in the pathogenesis of PMS.

We observed that subjects with PMS exhibited higher CES-D scores compared with those without PMS, both before and after menstruation (Table 3). This suggests that in patients with PMS, depressive tendency is related to PMS symptoms both before and after menstruation. Such a relationship may reflect an association between PMS and major depressive disorder.^22^ Indeed, there is some overlap between PMDD criteria, including insomnia and depression, and the CES-D scale. In addition, PMS is most often confused with depressive and anxiety disorders.^23^ These facts suggest that understanding the pathophysiology and treatment of psychiatric disorders may be useful for a better understanding of the background of PMS.

Previous studies have demonstrated increased oxidative stress in patients with depression.^24–26^ Biomarkers that have been shown to be relevant to depression include MDA and 8-OHdG.^27^ The increase in reactive oxygen species due to the activation of the sympathetic nervous system might lead to DNA oxidation in patients with depressive symptoms.^26^ These findings suggest that PMS and depression may be related to the DNA oxidative damage caused by oxidative stress. Therefore, regulating oxidative stress might help to control the pathogenesis of PMS. Duvan et al. reported that the total antioxidant capacity levels decreased on day 21 of the menstrual cycle; moreover, lipid hydroperoxide levels, which are indicators of oxidative status, significantly increased in subjects with PMS, while the oxidative balance shifted to the oxidant side.^9^

Our previous study indicated that the antioxidant capacity decreased before menstruation in subjects with PMS.^8^ Such phenomenon may reflect a change in the oxidant/antioxidant status and may be related to the pathogenesis of PMS. In this context, controlling the fluctuation of antioxidant capacity could be a promising strategy to cope with PMS.

Bellanti et al. suggested that both estrogens and progestin are potent antioxidants with protective and adaptive effects.^28^ In fact, estrogen level positively correlated with plasma antioxidant capacity and antioxidant enzyme expression throughout the menstrual cycle.^29–31^ These reports suggested that estrogen levels might influence the oxidant/antioxidant balance. Although estrogen levels were not measured in this study, they have likely fluctuated during the menstrual cycle. To confirm this, it is necessary to measure estrogen levels and to consider their associations with oxidative stress levels and antioxidant capacity.

To the best of our knowledge, there has been no previous study on the relationship between plasma 8-OHdG concentrations and symptoms and signs of PMS and PMDD in the same subjects. Our results indicate that 8-OHdG might be useful as an indicator of PMS. The measurement of 8-OHdG levels by blood sampling is more convenient than urine or tissue collection because it does not require sample concentration. In general, oxidative stress increases with age; since our subjects were young, the influence of age may not be significant.

This study had several limitations. First of all, our sample included female students of only one university; therefore, generalization of the findings may not be appropriate. Next, the sample size was rather small (*n* = 21), and PMS and PMDD were not diagnosed by prospective observation as recommended.^4^ Moreover, the luteal phase and follicular phase were not accurately determined. In addition, physical information such as height and weight was not collected. Finally, this study did not distinguish between premenstrual exacerbation and aggravation of an existing psychiatric or medical illness. To verify our findings, larger-scale studies in which PMS and PMDD are precisely diagnosed and follicular and luteal phases are identified are desirable.

## Conclusions

The increase in 8-OHdG was observed irrespective of premenstrual symptoms, suggesting that oxidative stress occurred before menstruation. Before menstruation, the concentration of 8-OHdG in subjects with PMS was higher than that in subjects without PMS, which indicates the presence of a premenstrual imbalance in oxidant/antioxidant status in subjects with PMS. To the best of our knowledge, this was the first study to compare 8-OHdG levels before menstruation with those after menstruation in the same subjects. Considering that the premenstrual imbalance in oxidant/antioxidant status may be involved in the pathogenesis of PMS, it may be a reasonable therapeutic target.

## Abbreviations Used

8-OHdG: 8-hydroxy-2′-deoxyguanosine
CES-D: Center for Epidemiologic Studies Depression
DSM-5: Diagnostic and Statistical Manual of Mental Disorders Fifth Edition
ECD: electrochemical detector
EDTA-2Na: ethylene diamine tetra-acetic acid disodium salt
HPLC: high-performance liquid chromatography
IQR: interquartile range
MDA: malondialdehyde
PMDD: premenstrual dysphoric disorder
PMS: premenstrual syndrome

## References

[1] Biggs WS, Demuth RH. Premenstrual syndrome and premenstrual dysphoric disorder. Am Fam Physician 2011;84:918–924. 22010771

[2] Baker LJ, Patrick B. Premenstrual syndrome (PMS): A peri-menopausal perspective. Maturitas 2012;72:121–125. 22534048 10.1016/j.maturitas.2012.03.007

[3] The International Statistical Classification of Diseases and Related Health Problems 10th Revision: World Health Organization, 2016.

[4] Association AP. Diagnostic and Statistical Manual of Mental Disorders: DSM-5. Arlington: American Psychiatric Association, 2013.

[5] Grady-Weliky TA. Clinical practice. Premenstrual dysphoric disorder. N Engl J Med 2003;348:433–438. 12556546 10.1056/NEJMcp012067

[6] Kelderhouse K, Taylor JS. A review of treatment and management modalities for premenstrual dysphoric disorder. Nurs Womens Health 2013;17:294–305. 23957795 10.1111/1751-486X.12048

[7] Machlin LJ. Use and safety of elevated dosages of vitamin E in adults. Int J Vitam Nutr Res Suppl 1989;30:56–68. 2507707

[8] Yama K, Minami E, Machida M, Hayase N, Miura J. Relation between premenstrual syndrome, oxidative stress and depression. Oyo Yakuri Pharmacometrics 2018;95:19–24.

[9] Duvan CI, Cumaoglu A, Turhan NO, Karasu C, Kafali H. Oxidant/antioxidant status in premenstrual syndrome. Arch Gynecol Obstet 2011;283:299–304. 20084389 10.1007/s00404-009-1347-y

[10] Kalia G, Sudheendran S, Rao A. Antioxidant status and lipid peroxidation in premenstrual syndrome: A preliminary study. Clin Chim Acta 2001;309:97–99. 11408011 10.1016/s0009-8981(01)00498-3

[11] Balat O, Dikensoy E, Ugur MG, Atmaca R, Cekmen M, Yurekli M. Malondialdehyde, nitrite and adrenomedullin levels in patients with premenstrual syndrome. Arch Gynecol Obstet 2007;275:361–365. 17063355 10.1007/s00404-006-0269-1

[12] Germadnik D, Pilger A, Rüdiger HW. Assay for the determination of urinary 8-hydroxy-2'-deoxyguanosine by high-performance liquid chromatography with electrochemical detection. J Chromatogr B Biomed Sci Appl 1997;689:399–403. 9080328 10.1016/s0378-4347(96)00328-3

[13] Sperati A, Abeni DD, Tagesson C, Forastiere F, Miceli M, Axelson O. Exposure to indoor background radiation and urinary concentrations of 8-hydroxydeoxyguanosine, a marker of oxidative DNA damage. Environ Health Perspect 1999;107:213–215. 10064551 10.1289/ehp.99107213 PMC1566395

[14] Black CN, Bot M, Scheffer PG, Cuijpers P, Penninx BW. Is depression associated with increased oxidative stress? A systematic review and meta-analysis. Psychoneuroendocrinology 2015;51:164–175. 25462890 10.1016/j.psyneuen.2014.09.025

[15] Alici D, Bulbul F, Virit O, et al. Evaluation of oxidative metabolism and oxidative DNA damage in patients with obsessive-compulsive disorder. Psychiatry Clin Neurosci 2016;70:109–115. 26388322 10.1111/pcn.12362

[16] Miyaoka Y, Akimoto Y, Ueda K, et al. Fulfillment of the premenstrual dysphoric disorder criteria confirmed using a self-rating questionnaire among Japanese women with depressive disorders. Biopsychosoc Med 2011;5:5. 21535889 10.1186/1751-0759-5-5 PMC3110105

[17] Steiner M, Macdougall M, Brown E. The premenstrual symptoms screening tool (PSST) for clinicians. Arch Womens Ment Health 2003;6:203–209. 12920618 10.1007/s00737-003-0018-4

[18] Inaba Y, Koide S, Yokoyama K, Karube I. Development of urinary 8-hydroxy-2'-deoxyguanosine (8-OHdG) measurement method combined with SPE. J Chromatogr Sci 2011;49:303–309. 21439122 10.1093/chrsci/49.4.303

[19] Koide S, Kinoshita Y, Ito N, Kimura J, Yokoyama K, Karube I. Determination of human serum 8-hydroxy-2'-deoxyguanosine (8-OHdG) by HPLC-ECD combined with solid phase extraction (SPE). J Chromatogr B Analyt Technol Biomed Life Sci 2010;878:2163–2167. 10.1016/j.jchromb.2010.06.015 20619753

[20] Radloff LS. The CES-D Scale: A self-report depression scale for research in the general population. Appl Psychol Meas 1977;1:385–401.

[21] Shima S, Shikano T, Kitamura T. A new self-report depression scale. Psychiatry 1985;27:717–723. (in Japanese).

[22] Forrester-Knauss C, Zemp SE, Weiss C, Tschudin S. The interrelation between premenstrual syndrome and major depression: Results from a population-based sample. BMC Public Health 2011;11:795. 21992230 10.1186/1471-2458-11-795 PMC3209462

[23] Facchinetti F, Tarabusi M, Nappi G. Premenstrual syndrome and anxiety disorders: A psychobiological link. Psychother Psychosom 1998;67:57–60. 9556195 10.1159/000012260

[24] Khanzode SD, Dakhale GN, Khanzode SS, Saoji A, Palasodkar R. Oxidative damage and major depression: The potential antioxidant action of selective serotonin re-uptake inhibitors. Redox Rep 2003;8:365–370. 14980069 10.1179/135100003225003393

[25] Arranz L, Guayerbas N, De la Fuente M. Impairment of several immune functions in anxious women. J Psychosom Res 2007;62:1–8. 17188114 10.1016/j.jpsychores.2006.07.030

[26] Iida T, Inoue K, Ito Y, et al. Comparison of urinary levels of 8-hydroxy-2'-deoxyguanosine between young females with and without depressive symptoms during different menstrual phases. Acta Med Okayama 2015;69:45–50. 25703170 10.18926/AMO/53121

[27] Lopresti AL, Maker GL, Hood SD, Drummond PD. A review of peripheral biomarkers in major depression: The potential of inflammatory and oxidative stress biomarkers. Prog Neuropsychopharmacol Biol Psychiatry 2014;48:102–111. 24104186 10.1016/j.pnpbp.2013.09.017

[28] Bellanti F, Matteo M, Rollo T, et al. Sex hormones modulate circulating antioxidant enzymes: Impact of estrogen therapy. Redox Biol 2013;1:340–346. 24024169 10.1016/j.redox.2013.05.003 PMC3757703

[29] Massafra C, Gioia D, De Felice C, et al. Effects of estrogens and androgens on erythrocyte antioxidant superoxide dismutase, catalase and glutathione peroxidase activities during the menstrual cycle. J Endocrinol 2000;167:447–452. 11115771 10.1677/joe.0.1670447

[30] Bednarek-Tupikowska G, Bohdanowicz-Pawlak A, Bidzińska B, Milewicz A, Antonowicz-Juchniewicz J, Andrzejak R. Serum lipid peroxide levels and erythrocyte glutathione peroxidase and superoxide dismutase activity in premenopausal and postmenopausal women. Gynecol Endocrinol 2001;15:298–303. 11560104

[31] Serviddio G, Loverro, G, Vicino, M, et al. Modulation of endometrial redox balance during the menstrual cycle: Relation with sex hormones. J Clin Endocrinol Metab 2002;87:2843–2848. 12050261 10.1210/jcem.87.6.8543

